# Crystal structure and Hirshfeld surface analysis of 1,4-di­aza­bicyclo­[2.2.2]octane­di­ium bis­(tribromide)

**DOI:** 10.1107/S2056989025009065

**Published:** 2025-10-28

**Authors:** Dmytro A. Haleliuk, Laurentiu Baltag, Dina D. Naumova, Valeriia O. Zozulia, Sofiia V. Partsevska

**Affiliations:** a Department of Chemistry, Taras Shevchenko National University of Kyiv, Volodymyrska str. 64/13, 01601 Kyiv, Ukraine; b ‘Petru Poni’ Institute of Macromolecular Chemistry, Aleea Grigore Ghica Voda 41-A, 700487 Iaşi, Romania; Institute of Chemistry, Chinese Academy of Sciences

**Keywords:** crystal structure, tribromide anion, Br⋯Br inter­actions, diprotonated DABCO

## Abstract

The crystal structure of the title salt, 1,4-di­aza­bicyclo­[2.2.2]octane­di­ium bis­(tribromide), (C_6_H_14_N_2_)[Br_3_]_2_, consists of diprotonated 1,4-di­aza­bicyclo­[2.2.2]octane­di­ium (or tri­ethyl­enedi­amine) cations, which are separated by [Br_3_]^−^ anions. Br⋯Br contacts between polybromide anions generate supra­molecular two-dimensional layers propagating along the *bc* plane. Organic cations are accommodated inside anionic layers and inter­act with tribromide anions through N—H⋯Br contacts. The structure is additionally stabilized by weak C—H⋯Br inter­actions linking the organic cations and [Br_3_]^−^. Hirshfeld surface analysis and associated fingerprint plots reveal that the main contributions to the crystal packing are provided by Br⋯H inter­actions (84.8%), followed by less chemically meaningful H⋯H (15.2%) contacts. As a novel example of a tribromide salt, the reported com­pound is of inter­est toward its potential use in organic synthesis as a regioselective brominating agent, in oxidation chemistry, and in materials processing involving metal dissolution and recovery.

## Abstract 2

The crystal structure of (C_6_H_14_N_2_)[Br_3_]_2_ contains diprotonated tri­ethyl­enediaminium cations, the charge of which is com­pensated by [Br_3_]^−^ anions. The crystal packing is stabilized by Br⋯Br contacts, forming two-dimensional layers, and by N—H⋯Br/C—H⋯Br inter­actions between cations and tribromide anions.

## 1. Chemical context

Halogens represent a highly adaptable group of elements with numerous significant applications. Among their distinctive features is the ability to generate inter­halogen species, as well as polyhalogen ions. Within this class, polyhalide anions – particularly those containing iodine – have been the subject of sustained inter­est for more than a century. A broad variety of these com­pounds has been identified and structurally characterized (Svensson & Kloo, 2003 ▸). Lighter halogens, however, tend to produce com­paratively fewer polyhalide anions, a trend largely attributed to their higher volatility relative to iodine. Nonetheless, several polybromide species have been reported since the first systematic study by Chattaway and Hoyle in 1923 (Chattaway & Hoyle, 1923 ▸), including monoanions ([Br_3_]^−^, [Br_5_]^−^, [Br_7_]^−^, [Br_9_]^−^ and [Br_11_]^−^) and dianions ([Br_4_]^2−^, [Br_6_]^2−^, [Br_8_]^2−^ and [Br_10_]^2−^) (Sonnenberg *et al.*, 2020 ▸).
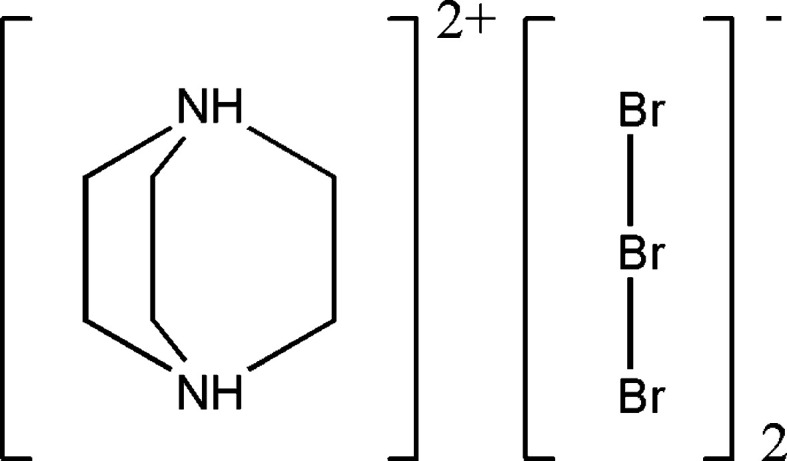


One of the most relevant applications of tribromide anions is their role as mild brominating reagents. In organic synthesis, tribromides serve as mild brominating agents with high regioselectivity, while nona­bromide anions, such as [NPr_4_][Br_9_], provide enhanced chemo- and stereoselectivity com­pared to Br_2_ (Beck *et al.*, 2014 ▸). Tribromide com­plexes have been successfully applied as selective oxidizing agents. For example, the [DBUH][Br_3_] com­plex (where DBUH = 1,8-di­aza­bicyclo­[5.4.0]undec-7-enium) was shown to promote efficient oxidations of sulfides to sulfoxides and of alcohols to carbonyl com­pounds under mild conditions, delivering high selectivity and avoiding over-oxidation (Bakavoli *et al.*, 2010*a* ▸, 2010*b* ▸). Polyhalide-based ionic liquids, such as [HMIM][Br_9_] (where HMIM = 1-hexyl-3-methyl­imidazolium), combine low viscosity with strong oxidizing ability (Sonnenberg *et al.*, 2020 ▸). These liquids have been successfully employed for the oxidative dissolution and recovery of metals and alloys (Van Den Bossche *et al.*, 2018 ▸).

In the present communication, we report on a new polybromide com­pound containing the tribromide anion, (C_6_H_14_N_2_)[Br_3_]_2_, its synthesis, crystal structure and Hirshfeld surface analysis.

## 2. Structural commentary

**Figure 1:**
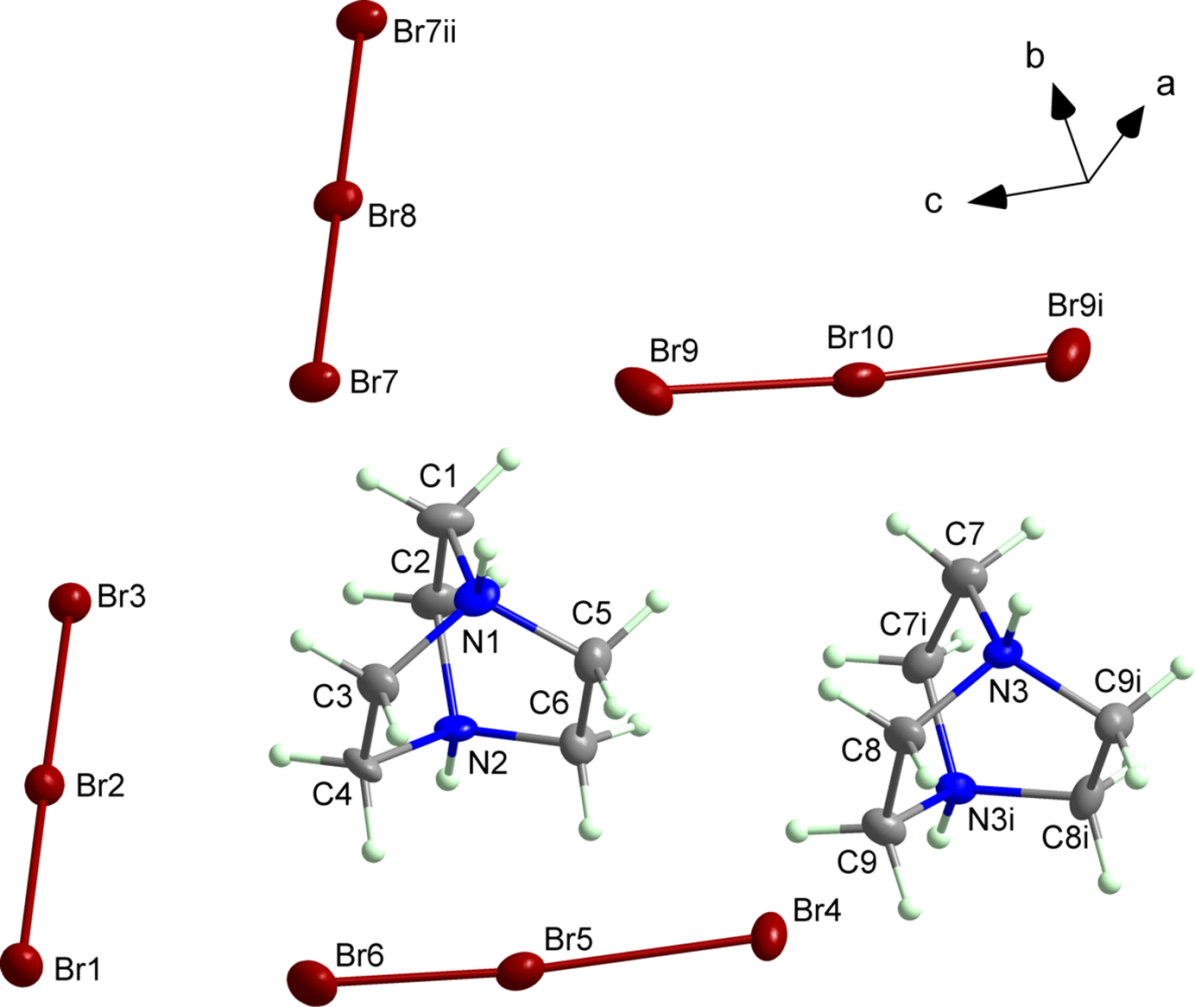
A fragment of the crystal structure of the title com­pound, showing the atom-labelling scheme. Displacement ellipsoids are drawn at the 50% probability level. [Symmetry codes: (i) −*x* + 1, *y*, −*z* + 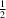; (ii) −*x* + 1, −*y* + 2, −*z* + 1.]

The title com­pound crystalizes in the monoclinic space group *C*2/*c*. It consists of diprotonated 1,4-di­aza­bicyclo­[2.2.2]octane­di­ium (or tri­ethyl­enedi­amine) cations separated by [Br_3_]^−^ anions (Fig. 1 ▸). The symmetry-independent unit contains 1.5 organic cation and9 Br atoms.

The Br—Br bond lengths are summarized in Table 1 ▸. Two symmetric and two asymmetric [Br_3_]^−^ anions are present in the structure. The Br4—Br5—Br6 anion exhibits two markedly different Br—Br distances, 2.8805 (9) and 2.3582 (9) Å, while in Br1—Br2—Br3, Br7—Br8—Br7^i^ and Br9—Br10—Br9^ii^, the bond lengths are similar (Table 1 ▸).

The symmetric tribromide anion is best described as a delocalized three-centre four-electron (3c-4e) unit (Br—Br—Br)^−^, in which the additional electron density is shared across the three Br atoms, giving peripheral Br—Br inter­actions of com­parable half-bond character. In contrast, an asymmetric tribromide anion exhibits localization of the bonding: one Br—Br distance is relatively short, approaching that of a conventional single/partial single bond, while the other is elongated and weak, essentially representing a halogen-bond-type contact rather than a typical covalent bond. Such asymmetric trihalides are well documented in the polyhalide literature, and structural surveys reveal a wide distribution of Br—Br distances (Pichierri, 2011 ▸).

The angles between the Br atoms lie in the range between 174.15 (3) (for Br4—Br5—Br6) and 180.00° (for Br7—Br8—Br7^i^) (Table 1 ▸). This range is also characteristic for other tribromide com­pounds listed in the *Database survey* section.

The charge of the tribromide anions is balanced by one fully independent asymmetric 1,4-di­aza­bicyclo­[2.2.2]octane­di­ium dication and one half of a symmetry-generated cation, both diprotonated. The C—C bond lengths fall in the range 1.530 (7)–1.545 (7) Å and the C—N bond lengths are 1.480 (7)–1.499 (7) Å. These values are typical for di­pro­ton­a­ted tri­ethyl­enedi­amine cations and are consistent with previous reports (Andrzejewski *et al.*, 2011 ▸).

## 3. Supra­molecular features

**Figure 2:**
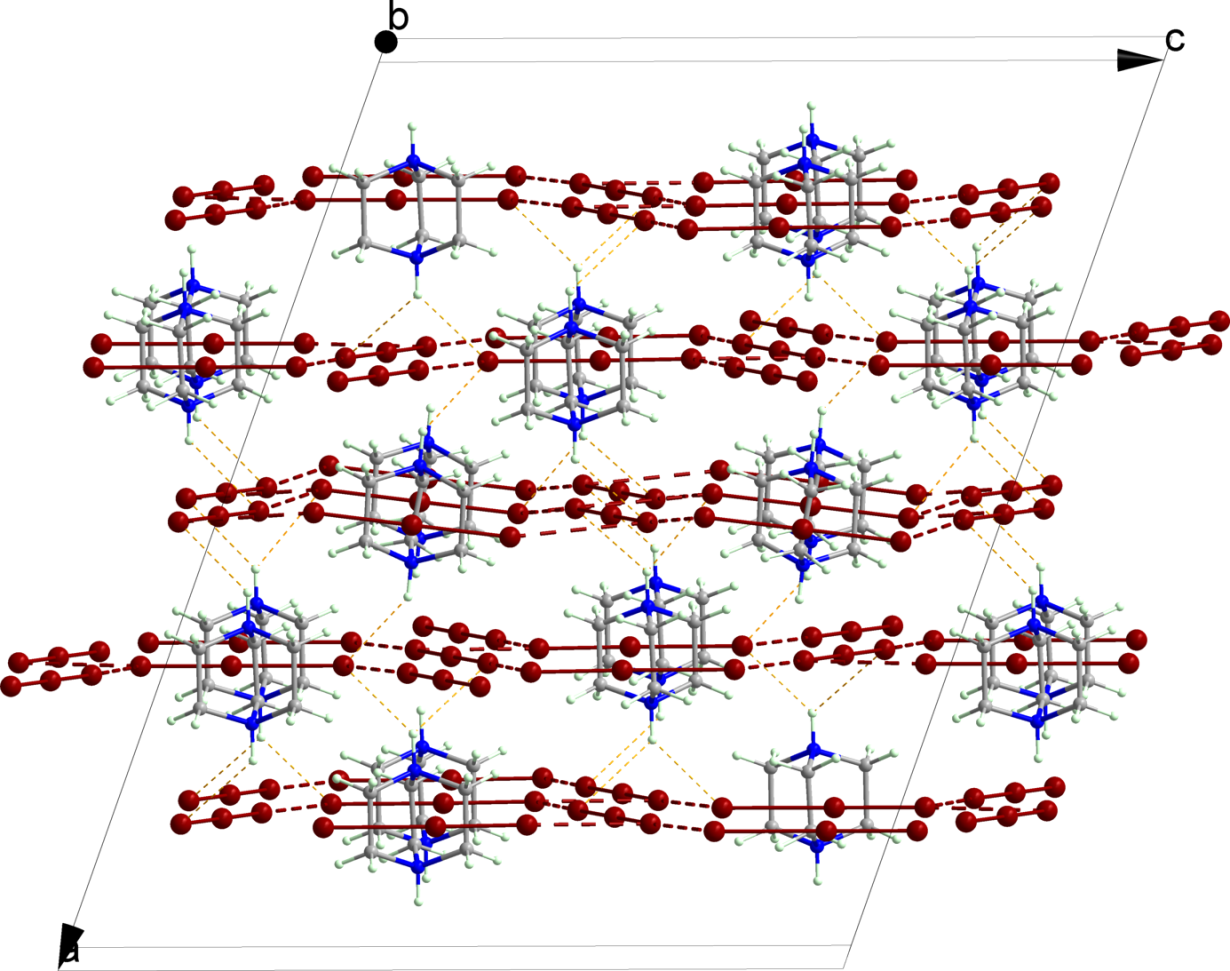
The crystal structure of the title com­pound in a view along the *bc* plane. Hydrogen bonds between organic cations and polybromide anions are shown as orange dashed lines. Br⋯Br contacts between [Br_3_]^−^ anions are shown as red dashed lines.

The polybromide anions form multiple inter­molecular Br⋯Br contacts ensuring the creation of supra­molecular two-dimensional layers which propagate along the *bc* plane (Fig. 2 ▸). All the supra­molecular Br⋯Br contacts (3.5378 Å on average) are smaller than the sum of the van der Waals radii of 3.7 Å for Br atoms. The organic cations are located inside anionic layers and are connected with tribromide anions through N—H⋯Br contacts (Fig. 2 ▸ and Table 2 ▸). In particular, some of the N—H⋯Br hy­dro­gen bonds exhibit significantly shorter H⋯Br distances com­pared to others. Such a close approach of the hy­dro­gen-bond donors to one end of the tribromide anion stabilizes charge localization, resulting in the elongation of one Br—Br bond and the shortening of the other. This effect is particularly pronounced for the Br4—Br5—Br6 anion, while its influence on the Br9—Br10—Br9^ii^ [symmetry code: (ii) −*x* + 1, *y*, −*z* + 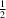] anion is less substantial.

In addition, there are weak C—H⋯Br contacts between the organic cations and [Br_3_]^−^ that provide additional stabilization of the structure.

## 4. Hirshfeld surface analysis

Hirshfeld surface analysis and two-dimensional fingerprint plots of the title com­pound were generated using *CrystalExplorer* (Spackman *et al.*, 2021 ▸).

**Figure 3:**
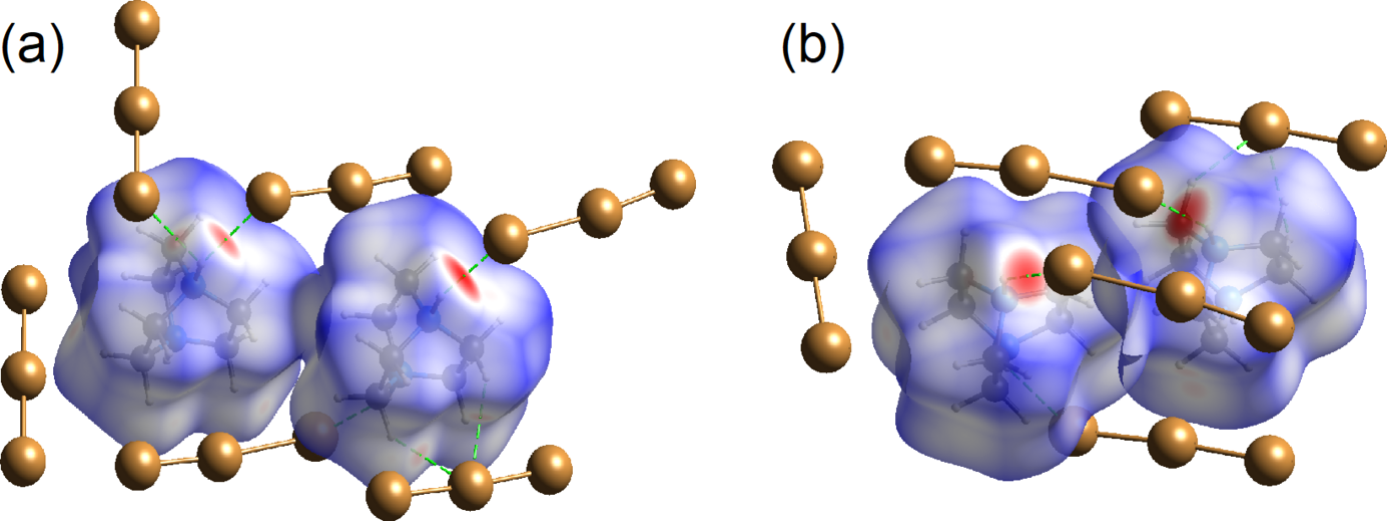
(*a*)/(*b*) Hirshfeld surface of the 1,4-di­aza­bicyclo­[2.2.2]octane­di­ium cation. Neighbouring Br atoms are shown in ball-and-stick mode for clarity. The surface regions with the strongest inter­molecular inter­actions are shown in red.

The Hirshfeld surface analysis of the 1,4-di­aza­bicyclo­[2.2.2]octane­di­ium cation highlights a pronounced N—H⋯Br hy­dro­gen bond with a neighbouring tribromide anion, visualized as an intense red spot (*d*_norm_ plot; Fig. 3 ▸).

**Figure 4:**
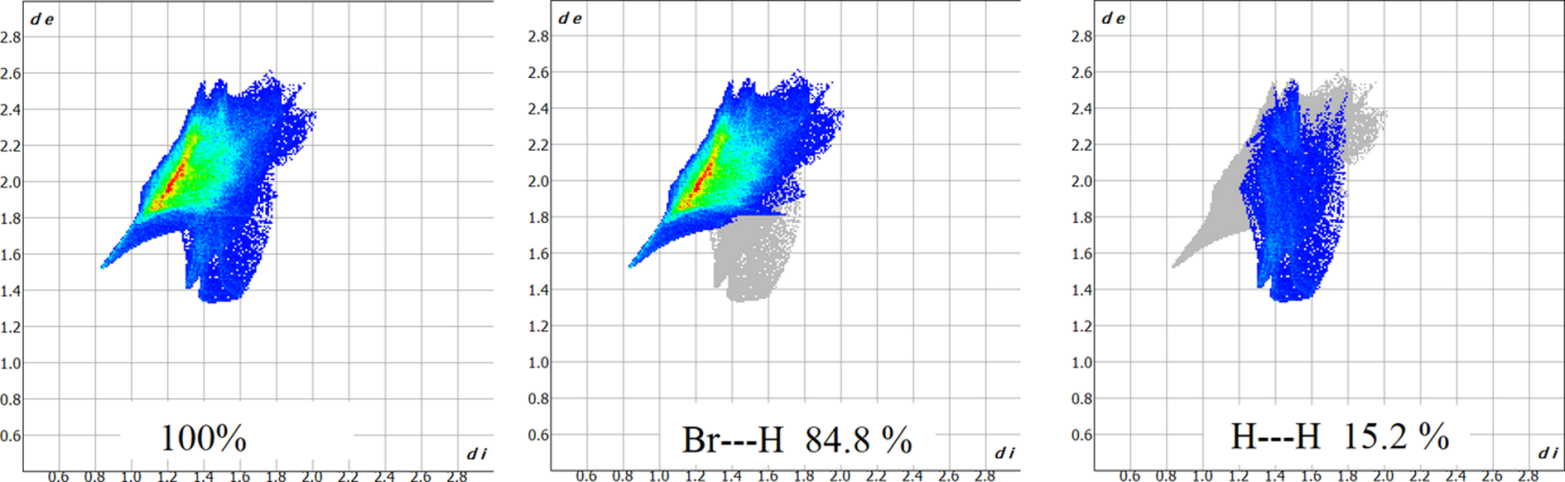
Fingerprint plots for 1,4-di­aza­bicyclo­[2.2.2]octane­di­ium, showing the overall (100%), Br⋯H (84.8%) and H⋯H (15.2%) contributions. The *d*_e_ and *d*_i_ values are the distances to the closest external and inter­nal atoms, respectively, from a given point to the Hirshfeld surface.

In addition, weaker C—H⋯Br contacts are observed. The relative contributions of these inter­actions to the crystal packing are depicted in the two-dimensional Hirshfeld fingerprint plots (Fig. 4 ▸).

Among them, Br⋯H inter­actions provide the largest contribution (84.8%), followed by H⋯H (15.2%) contacts, which, though originating from the terminal positions of H atoms, are chemically insignificant.

## 5. Database survey

A search of the tribromide anion in the Cambridge Structural Database (CSD, Version 6.00, last update April 2025; Groom *et al.*, 2016 ▸) revealed 312 crystal structures. A search of the monoprotonated DABCO cation revealed 375 crystal structures and a search of the diprotonated DABCO cation revealed 581 crystal structures. The closest analogues to the title com­pound were found to be bis­(1,4-diazo­niabi­cyclo­[2.2.2]octa­ne) bis­(1-aza-4-azoniabi­cyclo­[2.2.2]octa­ne) tetra­kis­(tri­bro­mide) dibromides [DAHGUO (Allwood *et al.*, 1985 ▸) and DAHGUO01 (Heravi *et al.*, 2005 ▸)], both of which contain the diprotonated DABCO cation. There is also quinuclidinium tribromide (REKBIS; Robertson *et al.*, 1997 ▸) with a similar bicyclic cation.

## 6. Synthesis and crystallization

DABCO (0.5 mmol) was mixed with PbBr_2_ (0.1 mmol) in 3 ml of HBr (48%). Bromine (1 mmol) was then added dropwise. The mixture was stirred for 4 h and the product was collected upon cooling of the solution. After filtration, light-orange crystals were obtained. They were separated and kept under Paratone oil until the diffraction experiment.

## 7. Refinement

Crystal data, data collection and structure refinement details are summarized in Table 3 ▸. Methyl­ene H atoms were positioned geometrically and refined with riding coordinates [*U*_iso_(H) = 1.2*U*_eq_(C)]. H atoms of the N—H groups were positioned geometrically and refined with riding coordinates and stretchable bonds [*U*_iso_(H) = 1.2*U*_eq_(N)].

## Supplementary Material

Crystal structure: contains datablock(s) global, I. DOI: 10.1107/S2056989025009065/nx2030sup1.cif

Structure factors: contains datablock(s) I. DOI: 10.1107/S2056989025009065/nx2030Isup2.hkl

CCDC reference: 2495993

Additional supporting information:  crystallographic information; 3D view; checkCIF report

## Figures and Tables

**Table 1:** Geometric paramerets of polybromide anions (Å, °)

	Br—Br	Br—Br—Br
Br1—Br2—Br3	2.5429 (9); 2.5780 (9)	179.51 (3)
Br4—Br5—Br6	2.8805 (9); 2.3582 (9)	174.15 (3)
Br7—Br8—Br7^i^	2.5612 (6)	180
Br9—Br10—Br9^ii^	2.5405 (7)	175.78 (5)

Symmetry codes: (i) −*x* + 1, −*y* + 2, −*z* + 1; (ii) −*x* + 1, *y*, −*z* + 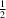.

**Table 2:** Hydrogen-bond geometry (Å, °)

*D*—H⋯*A*	*D*—H	H⋯*A*	*D*⋯*A*	*D*—H⋯*A*
N1—H1⋯Br7	0.89 (5)	2.87 (2)	3.491 (4)	128 (1)
N1—H1⋯Br9	0.89 (5)	2.66 (5)	3.386 (5)	138 (3)
N2—H2⋯Br3^i^	0.95 (5)	2.90 (4)	3.526 (5)	125 (1)
N2—H2⋯Br4^ii^	0.95 (5)	2.51 (2)	3.256 (4)	136 (2)
N3—H3⋯Br4^iii^	0.89 (5)	2.46 (5)	3.209 (5)	142 (3)

Symmetry codes: (i) 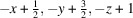; (ii) 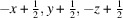; (iii) 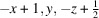.

**Table 3:** Experimental details

Crystal data	
Chemical formula	C_6_H_14_N_2_^2+^·2Br_3_^−^
*M*_r_	593.65
Crystal system, space group	Monoclinic, *C*2/*c*
Temperature (K)	180
*a*, *b*, *c* (Å)	24.7979 (7), 8.9860 (2), 20.1594 (6)
β (°)	109.675 (3)
*V* (Å^3^)	4229.9 (2)
*Z*	12
Radiation type	Mo *K*α
μ (mm^−1^)	17.06
Crystal size (mm)	0.19 × 0.13 × 0.1

Data collection	
Diffractometer	Rigaku Xcalibur Eos
Absorption correction	Analytical (*CrysAlis PRO*; Rigaku OD, 2024 ▸)
*T*_min_, *T*_max_	0.101, 0.276
No. of measured, independent and observed [*I* > 2σ(*I*)] reflections	18451, 5033, 3541
*R*_int_	0.062
(sin θ/λ)_max_ (Å^−1^)	0.693

Refinement	
*R*[*F*^2^ > 2σ(*F*^2^)], *wR*(*F*^2^), *S*	0.049, 0.061, 1.04
No. of reflections	5033
No. of parameters	196
H-atom treatment	H-atom parameters constrained
Δρ_max_, Δρ_min_ (e Å^−3^)	0.85, −0.83

Computer programs: *CrysAlis PRO* (Rigaku OD, 2024 ▸), *SHELXT2018* (Sheldrick, 2015*a* ▸), *SHELXL2019* (Sheldrick, 2015*b* ▸) and *OLEX2* (Dolomanov *et al.*, 2009 ▸).

## supplementary crystallographic information

### 1,4-Diazabicyclo[2.2.2]octanediium bis(tribromide) Crystal data

**Table:**

C_6_H_14_N_2_^2+^·2Br_3_^−^	*F*(000) = 3288
*M**_r_* = 593.65	*D*_x_ = 2.797 Mg m^−^^3^
Monoclinic, *C*2/*c*	Mo *K*α radiation, λ = 0.71073 Å
*a* = 24.7979 (7) Å	Cell parameters from 3922 reflections
*b* = 8.9860 (2) Å	θ = 2.3–28.1°
*c* = 20.1594 (6) Å	µ = 17.06 mm^−^^1^
β = 109.675 (3)°	*T* = 180 K
*V* = 4229.9 (2) Å^3^	Prism, clear light orange
*Z* = 12	0.19 × 0.13 × 0.1 mm

### 1,4-Diazabicyclo[2.2.2]octanediium bis(tribromide) Data collection

**Table:**

Rigaku Xcalibur Eos diffractometer	5033 independent reflections
Radiation source: fine-focus sealed X-ray tube, Enhance (Mo) X-ray Source	3541 reflections with *I* > 2σ(*I*)
Graphite monochromator	*R*_int_ = 0.062
Detector resolution: 16.1593 pixels mm^-1^	θ_max_ = 29.5°, θ_min_ = 1.7°
ω scans	*h* = −32→33
Absorption correction: analytical (CrysAlis PRO; Rigaku OD, 2024)	*k* = −11→11
*T*_min_ = 0.101, *T*_max_ = 0.276	*l* = −26→27
18451 measured reflections	

### 1,4-Diazabicyclo[2.2.2]octanediium bis(tribromide) Refinement

**Table:**

Refinement on *F*^2^	Hydrogen site location: inferred from neighbouring sites
Least-squares matrix: full	H-atom parameters constrained
*R*[*F*^2^ > 2σ(*F*^2^)] = 0.049	*w* = 1/[σ^2^(*F*_o_^2^) + (0.0062*P*)^2^] where *P* = (*F*_o_^2^ + 2*F*_c_^2^)/3
*wR*(*F*^2^) = 0.061	(Δ/σ)_max_ = 0.001
*S* = 1.04	Δρ_max_ = 0.85 e Å^−^^3^
5033 reflections	Δρ_min_ = −0.83 e Å^−^^3^
196 parameters	Extinction correction: SHELXL2019 (Sheldrick, 2015*b*), Fc^*^=kFc[1+0.001xFc^2^λ^3^/sin(2θ)]^-1/4^
0 restraints	Extinction coefficient: 0.000083 (3)
Primary atom site location: dual	

### 1,4-Diazabicyclo[2.2.2]octanediium bis(tribromide) Special details

**Table:**

Geometry. All esds (except the esd in the dihedral angle between two l.s. planes) are estimated using the full covariance matrix. The cell esds are taken into account individually in the estimation of esds in distances, angles and torsion angles; correlations between esds in cell parameters are only used when they are defined by crystal symmetry. An approximate (isotropic) treatment of cell esds is used for estimating esds involving l.s. planes.

### 1,4-Diazabicyclo[2.2.2]octanediium bis(tribromide) Fractional atomic coordinates and isotropic or equivalent isotropic displacement parameters (Å^2^)

**Table:**

	*x*	*y*	*z*	*U*_iso_*/*U*_eq_	
Br2	0.33244 (2)	0.50575 (7)	0.64045 (3)	0.02256 (15)	
Br3	0.33170 (2)	0.77017 (7)	0.59054 (3)	0.02278 (15)	
Br10	0.500000	0.60990 (9)	0.250000	0.0252 (2)	
Br5	0.33242 (2)	0.11522 (7)	0.41626 (3)	0.02463 (16)	
Br4	0.33268 (2)	0.10617 (7)	0.27353 (3)	0.02476 (16)	
Br8	0.500000	1.000000	0.500000	0.0249 (2)	
Br1	0.33315 (3)	0.24402 (7)	0.68869 (3)	0.02580 (16)	
Br7	0.50095 (2)	0.73650 (7)	0.54881 (3)	0.02625 (16)	
Br6	0.33155 (3)	0.14927 (7)	0.53188 (3)	0.03047 (17)	
Br9	0.51368 (3)	0.62030 (8)	0.38060 (3)	0.03385 (18)	
N2	0.28257 (18)	0.5789 (5)	0.3643 (2)	0.0167 (11)	
H2	0.242 (2)	0.5785 (5)	0.3461 (9)	0.020*	
N3	0.55301 (19)	0.0763 (5)	0.2665 (2)	0.0167 (11)	
H3	0.591 (2)	0.0763 (5)	0.2778 (7)	0.020*	
N1	0.38870 (18)	0.5790 (5)	0.4120 (2)	0.0194 (12)	
H1	0.427 (2)	0.5794 (5)	0.4287 (9)	0.023*	
C4	0.3012 (2)	0.5139 (6)	0.4374 (3)	0.0180 (14)	
H4A	0.286087	0.411521	0.435981	0.022*	
H4B	0.286299	0.575026	0.468271	0.022*	
C3	0.3673 (2)	0.5120 (7)	0.4661 (3)	0.0230 (15)	
H3A	0.381497	0.569881	0.510404	0.028*	
H3B	0.381323	0.408464	0.476170	0.028*	
C8	0.5380 (2)	0.0047 (6)	0.3243 (3)	0.0217 (14)	
H8A	0.552330	0.065723	0.367606	0.026*	
H8B	0.555898	−0.094974	0.334504	0.026*	
C6	0.3041 (2)	0.4874 (7)	0.3175 (3)	0.0219 (14)	
H6A	0.289743	0.527505	0.268904	0.026*	
H6B	0.290410	0.383638	0.316405	0.026*	
C9	0.4724 (2)	−0.0094 (6)	0.3011 (3)	0.0225 (15)	
H9A	0.461046	−0.115398	0.293658	0.027*	
H9B	0.458416	0.031316	0.337972	0.027*	
C1	0.3681 (2)	0.7365 (7)	0.3979 (3)	0.0280 (16)	
H1A	0.384404	0.783387	0.364353	0.034*	
H1B	0.380795	0.794392	0.442216	0.034*	
C7	0.53241 (19)	0.2334 (7)	0.2562 (3)	0.0232 (15)	
H7A	0.539921	0.277607	0.215221	0.028*	
H7B	0.552795	0.293208	0.298496	0.028*	
C2	0.3027 (2)	0.7363 (6)	0.3668 (3)	0.0244 (15)	
H2A	0.286432	0.797200	0.396380	0.029*	
H2B	0.290034	0.779202	0.318716	0.029*	
C5	0.3697 (2)	0.4911 (6)	0.3457 (3)	0.0224 (15)	
H5A	0.385023	0.388602	0.354820	0.027*	
H5B	0.384278	0.537359	0.310429	0.027*	

### 1,4-Diazabicyclo[2.2.2]octanediium bis(tribromide) Atomic displacement parameters (Å^2^)

**Table:**

	*U*^11^	*U*^22^	*U*^33^	*U*^12^	*U*^13^	*U*^23^
Br2	0.0212 (3)	0.0242 (4)	0.0228 (3)	−0.0002 (2)	0.0080 (3)	−0.0020 (3)
Br3	0.0228 (3)	0.0212 (4)	0.0239 (3)	0.0001 (3)	0.0073 (3)	−0.0004 (3)
Br10	0.0196 (5)	0.0144 (5)	0.0410 (6)	0.000	0.0095 (4)	0.000
Br5	0.0242 (3)	0.0164 (4)	0.0312 (4)	−0.0003 (3)	0.0066 (3)	−0.0011 (3)
Br4	0.0168 (3)	0.0360 (4)	0.0208 (3)	0.0009 (3)	0.0055 (3)	−0.0078 (3)
Br8	0.0213 (5)	0.0226 (5)	0.0258 (5)	−0.0005 (4)	0.0011 (4)	0.0001 (4)
Br1	0.0301 (4)	0.0237 (4)	0.0251 (4)	0.0009 (3)	0.0112 (3)	0.0023 (3)
Br7	0.0259 (4)	0.0223 (4)	0.0255 (4)	−0.0020 (3)	0.0019 (3)	0.0036 (3)
Br6	0.0344 (4)	0.0294 (4)	0.0271 (4)	−0.0008 (3)	0.0097 (3)	0.0086 (3)
Br9	0.0235 (4)	0.0420 (5)	0.0351 (4)	0.0002 (3)	0.0086 (3)	0.0158 (4)
N2	0.010 (2)	0.015 (3)	0.022 (3)	−0.0036 (19)	0.002 (2)	0.001 (2)
N3	0.013 (2)	0.014 (3)	0.023 (3)	−0.001 (2)	0.006 (2)	−0.002 (2)
N1	0.009 (2)	0.021 (3)	0.022 (3)	0.002 (2)	−0.003 (2)	0.000 (2)
C4	0.013 (3)	0.026 (4)	0.016 (3)	−0.003 (2)	0.006 (3)	0.007 (3)
C3	0.024 (4)	0.026 (4)	0.018 (3)	−0.001 (3)	0.005 (3)	0.005 (3)
C8	0.025 (4)	0.022 (4)	0.017 (3)	−0.003 (3)	0.005 (3)	0.005 (3)
C6	0.018 (3)	0.025 (4)	0.023 (4)	0.002 (3)	0.009 (3)	−0.002 (3)
C9	0.021 (3)	0.022 (4)	0.026 (4)	0.000 (3)	0.010 (3)	0.005 (3)
C1	0.021 (4)	0.020 (4)	0.040 (4)	−0.003 (3)	0.007 (3)	0.005 (3)
C7	0.024 (3)	0.020 (4)	0.023 (4)	0.000 (3)	0.005 (3)	0.004 (3)
C2	0.027 (4)	0.014 (4)	0.030 (4)	0.000 (3)	0.008 (3)	0.002 (3)
C5	0.022 (3)	0.025 (4)	0.020 (3)	0.003 (3)	0.006 (3)	−0.003 (3)

### 1,4-Diazabicyclo[2.2.2]octanediium bis(tribromide) Geometric parameters (Å, º)

**Table:**

Br2—Br3	2.5780 (9)	C4—C3	1.545 (7)
Br2—Br1	2.5429 (9)	C3—H3A	0.9900
Br10—Br9	2.5405 (7)	C3—H3B	0.9900
Br10—Br9^i^	2.5405 (7)	C8—H8A	0.9900
Br5—Br4	2.8805 (9)	C8—H8B	0.9900
Br5—Br6	2.3582 (9)	C8—C9	1.539 (7)
Br8—Br7	2.5612 (6)	C6—H6A	0.9900
Br8—Br7^ii^	2.5612 (6)	C6—H6B	0.9900
N2—H2	0.95 (5)	C6—C5	1.533 (7)
N2—C4	1.505 (6)	C9—H9A	0.9900
N2—C6	1.480 (7)	C9—H9B	0.9900
N2—C2	1.496 (7)	C1—H1A	0.9900
N3—H3	0.88 (5)	C1—H1B	0.9900
N3—C8	1.485 (6)	C1—C2	1.530 (7)
N3—C9^i^	1.506 (6)	C7—C7^i^	1.541 (9)
N3—C7	1.491 (7)	C7—H7A	0.9900
N1—H1	0.89 (5)	C7—H7B	0.9900
N1—C3	1.491 (7)	C2—H2A	0.9900
N1—C1	1.499 (7)	C2—H2B	0.9900
N1—C5	1.486 (6)	C5—H5A	0.9900
C4—H4A	0.9900	C5—H5B	0.9900
C4—H4B	0.9900		
			
Br1—Br2—Br3	179.5	Br7—Br8—Br7	180.0
Br4—Br5—Br6	174.2	Br9—Br10—Br9	175.8
			
N2—C4—C3—N1	1.6 (6)	C6—N2—C2—C1	−62.5 (6)
N2—C6—C5—N1	2.8 (6)	C9^i^—N3—C8—C9	−55.4 (5)
N3—C8—C9—N3^i^	−9.0 (6)	C9^i^—N3—C7—C7^i^	66.9 (7)
N1—C1—C2—N2	2.2 (7)	C1—N1—C3—C4	60.6 (5)
C4—N2—C6—C5	−63.0 (6)	C1—N1—C5—C6	−62.4 (6)
C4—N2—C2—C1	60.4 (6)	C7—N3—C8—C9	66.5 (5)
C3—N1—C1—C2	−63.4 (6)	C2—N2—C4—C3	−62.8 (5)
C3—N1—C5—C6	59.5 (6)	C2—N2—C6—C5	59.7 (6)
C8—N3—C7—C7^i^	−55.3 (7)	C5—N1—C3—C4	−61.7 (6)
C6—N2—C4—C3	60.4 (5)	C5—N1—C1—C2	59.4 (6)

Symmetry codes: (i) −*x*+1, *y*, −*z*+1/2; (ii) −*x*+1, −*y*+2, −*z*+1.

### 1,4-Diazabicyclo[2.2.2]octanediium bis(tribromide) Hydrogen-bond geometry (Å, º)

**Table:**

*D*—H···*A*	*D*—H	H···*A*	*D*···*A*	*D*—H···*A*
N1—H1···Br7	0.89 (5)	2.87 (2)	3.491 (4)	128 (1)
N1—H1···Br9	0.89 (5)	2.66 (5)	3.386 (5)	138 (3)
N2—H2···Br3^iii^	0.95 (5)	2.90 (4)	3.526 (5)	125 (1)
N2—H2···Br4^iv^	0.95 (5)	2.51 (2)	3.256 (4)	136 (2)
N3—H3···Br4^i^	0.89 (5)	2.46 (5)	3.209 (5)	142 (3)

Symmetry codes: (i) −*x*+1, *y*, −*z*+1/2; (iii) −*x*+1/2, −*y*+3/2, −*z*+1; (iv) −*x*+1/2, *y*+1/2, −*z*+1/2.
